# 

**Published:** 1887-12

**Authors:** 


Send in Your Subscription for 1888.

See Prospectus inside for Account of Premiums.

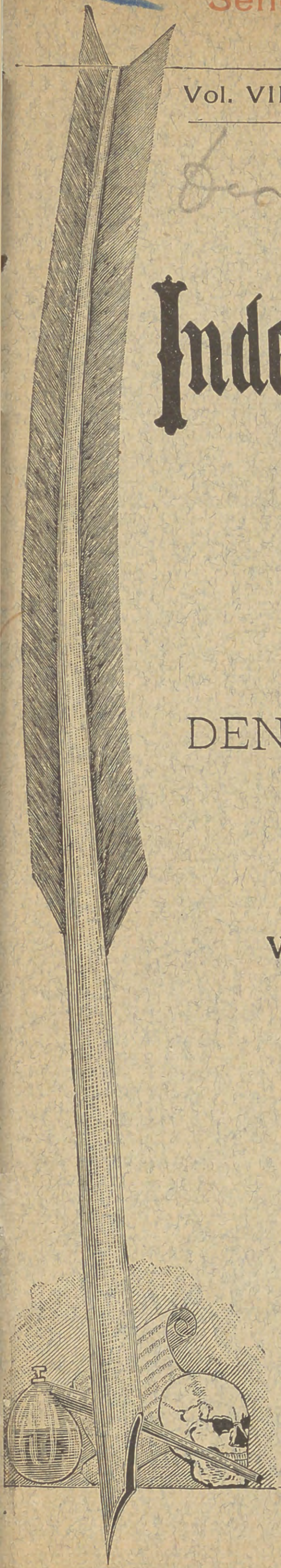

Vol. VIII. DECEMBER, 1887. No. 12.

LIBRARY
INDEXED
S.G.O.

### THE Independent Practitioner:

### A Monthly Journal

DEVOTED TO

### DENTAL AND ORAL SCIENCE.

---

**W. C. BARRETT, M. D., D. D. S.,**
EDITOR.

---

The New York Dental Journal Association,
PUBLISHERS,
No. 35 West 46th Street, New York.
AND
No. 208 Franklin St., Buffalo, N. Y.

**$2.50 Per Annum in Advance, . . . . Single Copies, 25 Cents.**

Entered at the Post-Office, Buffalo, N. Y., as Second-Class matter.

